# A highly conserved amino acid in VP1 regulates maturation of enterovirus 71

**DOI:** 10.1371/journal.ppat.1006625

**Published:** 2017-09-22

**Authors:** Yong-Xin Zhang, Yu-Ming Huang, Quan-Jie Li, Xiao-Yu Li, Yong-Dong Zhou, Fei Guo, Jin-Ming Zhou, Shan Cen

**Affiliations:** 1 Institute of Medicinal Biotechnology, Chinese Academy of Medical Sciences and Peking Union Medical School, Beijing, PR China; 2 Beijing Ditan Hospital, Capital Medical University, Beijing, China; 3 National Institute for Viral Disease Control and Prevention, China CDC, Beijing, China; 4 Institute of Pathogen Biology, Chinese Academy of Medical Sciences and Peking Union Medical School, Beijing, PR China; University of Pittsburgh, UNITED STATES

## Abstract

Enterovirus 71 (EV71) is the major causative agent of hand, foot and mouth disease (HFMD) in children, causing severe clinical outcomes and even death. Here, we report an important role of the highly conserved alanine residue at position 107 in the capsid protein VP1 (VP1^A107^) in the efficient replication of EV71. Substitutional mutations of VP1^A107^ significantly diminish viral growth kinetics without significant effect on viral entry, expression of viral genes and viral production. The results of mechanistic studies reveal that VP1^A107^ regulates the efficient cleavage of the VP0 precursor during EV71 assembly, which is required, in the next round of infection, for the transformation of the mature virion (160S) into an intermediate or A-particle (135S), a key step of virus uncoating. Furthermore, the results of molecular dynamic simulations and hydrogen-bond networks analysis of VP1^A107^ suggest that flexibility of the VP1 BC loop or the region surrounding the VP1^107^ residue directly correlates with viral infectivity. It is possible that sufficient flexibility of the region surrounding the VP1^107^ residue favors VP0 conformational change that is required for the efficient cleavage of VP0 as well as subsequent viral uncoating and viral replication. Taken together, our data reveal the structural role of the highly conserved VP1^A107^ in regulating EV71 maturation. Characterization of this novel determinant of EV71 virulence would promote the study on pathogenesis of Enteroviruses.

## Author summary

Enterovirus 71 (EV71) causes hand, foot and mouth disease in children. In this work, we have discovered, for the first time, that the alanine residue at position 107 of the capsid protein VP1 (VP1^A107^), which is present in almost all EV71 strains, plays an important role in the replication of EV71, since mutation of VP1^A107^ significantly impairs viral growth. Detailed mechanistic studies reveal that VP1^A107^ determines the efficient cleavage of the VP0 precursor during EV71 assembly, and subsequent virion uncoating in the next round of viral infection. We also observed that flexibility of the VP1 BC loop or the VP1^A107^ region directly associates with viral infectivity. Collectively, our data suggest a structural role of the highly conserved VP1^A107^ residue in regulating viral maturation, which is instrumental for the viral uncoating and viral replication. Discovery of this novel determinant of EV71 virulence would promote the study on pathogenesis of Enteroviruses.

## Introduction

Enterovirus 71 (EV71) is a small, non-enveloped, positive-stranded RNA virus, a member of the human enterovirus species A in the Enterovirus genus, the Picornaviridae family [1]. This virus is the major causative agent of hand, foot and mouth disease (HFMD), a febrile illness that commonly affects young children. Although often mild and self-limiting, EV71 infection may cause severe diseases including poliomyelitis-like paralysis, brainstem encephalitis and fatal cardiorespiratory failure [2–4]. In recent decades, EV71 outbreaks in the Asia-Pacific region have impacted millions of children, and have caused thousands of deaths [5–8].

The EV71 genome is protected by an icosahedrally symmetric capsid that is made of 60 copies of viral capsid proteins VP1 and VP3 and an average of 58 to 59 copies of capsid proteins VP2 and VP4, in addition to 1 to 2 copies of their capsid precursor protein VP0. During virus assembly, the viral capsid precursor P1 is firstly cleaved to yield VP0 (36 kDa), VP1 (32 kDa) and VP3 (27 kDa), forming a heterotrimer protomer. Five protomers then join together to form the pentameric assembly subunit, twelve of which self-assemble into an empty particle named as procapsid [9, 10]. Procapsids comprise 60 copies of VP0, VP1 and VP3, can be sedimentated as an 80S complex [11]. Packaging of the genomic RNA into the procapsid leads to the formation of the provirion concurrent with the cleavage of VP0 into VP2 (28 kDa) and VP4 (8 kDa), resulting in the formation of the mature virions that are sedimentated as 160S complexes [12]. VP0 cleavage, also called maturation cleavage, is hypothesized to be an autocatalytic event that results from the interactions between the structural proteins and the RNA genome during viral RNA encapsidation. This process is essential for virion stability and infectivity [13, 14].

Structural studies have revealed the processes that occur during enterovirus infection of cells. Cellular receptors first attach to the virus capsids, often binding to a canyon-like cavity surrounding the five-fold axis [15, 16]. This interaction triggers conformational changes in the viral capsid, resulting in the formation of an expanded intermediate particle, referred to as A-particle with a sediment value of 135S [17]. During the expansion process, the N terminus of VP1 is externalized, and VP4 is expelled from the capsid [18, 19]. These changes poise the particle for genome release [20]. Subsequently, a second and unknown trigger causes the genome to egress via a channel near the icosahedral two-fold axis of symmetry, leaving behind an empty capsid shell containing VP1, VP2 and VP3, with a sediment value of about 80S [17]. This series of conformational changes during the uncoating process imply that the cleavage efficiency of VP0 into VP2 and VP4 determines the conversion of A-particles from the mature virions as well as the subsequent release of viral genomic RNA.

EV71 is genetically diverse. For example, the VP1 gene undergoes an estimated mutation rate of 4.2–4.5×10^−3^ substitutions per site year^-1^ [21]. In addition, recombination events also play an important role in the emergence of EV71 subgenogroups with different virulence potentials and disease associations [22, 23]. In our previous work, we isolated the EV71-HP strain that belongs to genotype C4, from a HFMD patient with severe neurological complications during the 2008 EV71 epidemic in Anhui province of South China. By continuous multiple passages of this HP strain in Vero cells, we isolated the cell adapted strain, named EV71-CCA. We also found that growth of the CCA strain is significantly impaired in cell culture compared to the parental strain HP. In this study, we have further characterized the CCA strain and identified the genetic determinant behind the attenuated virulence of the CCA strain. A single amino acid alanine at position 107 in the VP1 protein, when changed to threonine, in the CCA strain, dramatically diminishes EV71 replication as a result of impaired maturation cleavage of the VP0 protein during EV71 assembly and subsequent viral uncoating.

## Results

### EV71-CCA exhibits slower replication kinetics compared with the parental strain EV71-HP

**Fig 1:**
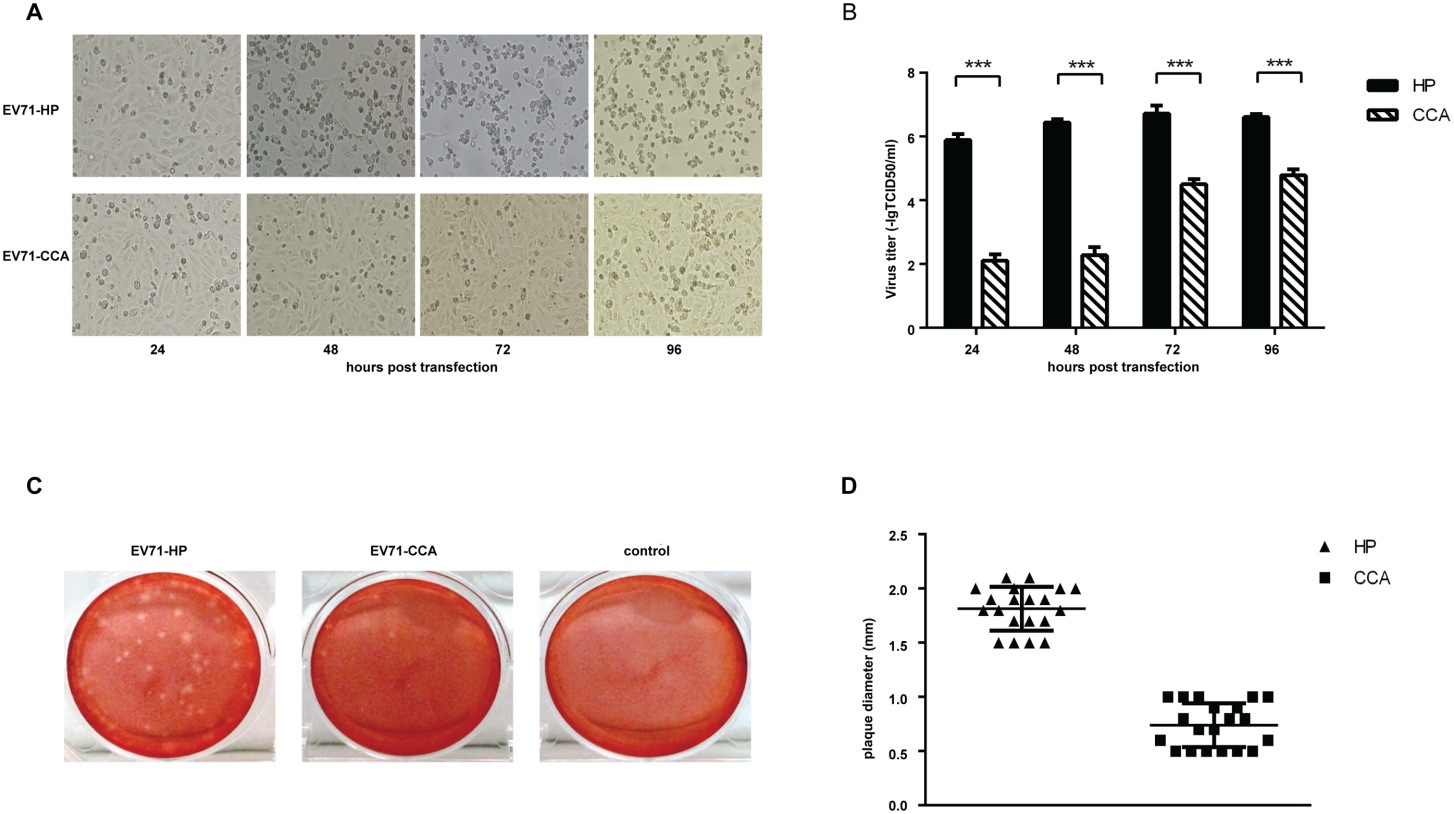
Characterization of EV71-HP and EV71-CCA. (A) Comparison of the cytopathic effects induced by transfection of the genomic RNA of EV71. CPE was monitored at 24, 48, 72 and 96 hours post transfection. (B) Growth kinetics of the two EV71 strains in Vero cells. Virions were collected at the indicated times and then titrated in theTCID_50_ assay. All experiments were performed in triplicates. At each time point, titers are means of three samples. Error bars represent SEM. (C) Plaque phenotypes of EV71-HP and EV71-CCA. Infected cells were incubated for 7 days before staining with 0.01% neutral red. (D) Comparison of plaque sizes of EV71-HP and EV71-CCA. Mean plaque size was determined by sampling 20 plaques.

We first examined the replication capability of both EV71-HP and EV71-CCA strains by comparing their growth kinetics and plaque formation in Vero cells. After transfecting Vero cells with equal amounts of *in vitro* transcribed full-length viral RNA, both strains were able to induce cytopathic effect (CPE) 24 hours after transfection. However, a significant delay was observed for EV71-CCA to cause CPE compared with EV71-HP. For example, CPE was observed in 100% of Vero cells 3 days after transfection with the EV71-HP RNA (Fig 1A), whereas it took 7 days for the EV71-CCA strain to cause CPE in all cells (S1 Fig). This suggests a lower infectivity of EV71-CCA than EV71-HP. When the titers of viruses were determined in plaque assays, transfection of the EV71-HP RNA produced approximate 10^4^ fold more infectious viruses than EV71-CCA at 24 and 48 hours post transfection (Fig 1B). This difference gradually diminished at later time points (Fig 1B). In addition, EV71-CCA displayed a pinpoint-plaque phenotype, whereas EV71-HP exhibited a large-plaque phenotype after 7 days of infection (Fig 1C). The mean plaque size of both strains was determined by sampling 20 plaques for each virus, and the results showed that the mean diameter of EV71-HP plaques is approximately 2-fold greater than that of EV71-CCA plaques (Fig 1D). Taken together, these data demonstrate a significant defect in the replication of EV71-CCA compared to EV71-HP.

### The different growth capacities of viruses EV71-HP and EV71-CCA are determined by a viral genome fragment containing genes *vp1*, *vp2*, *vp3* or *vp4*

**Fig 2:**
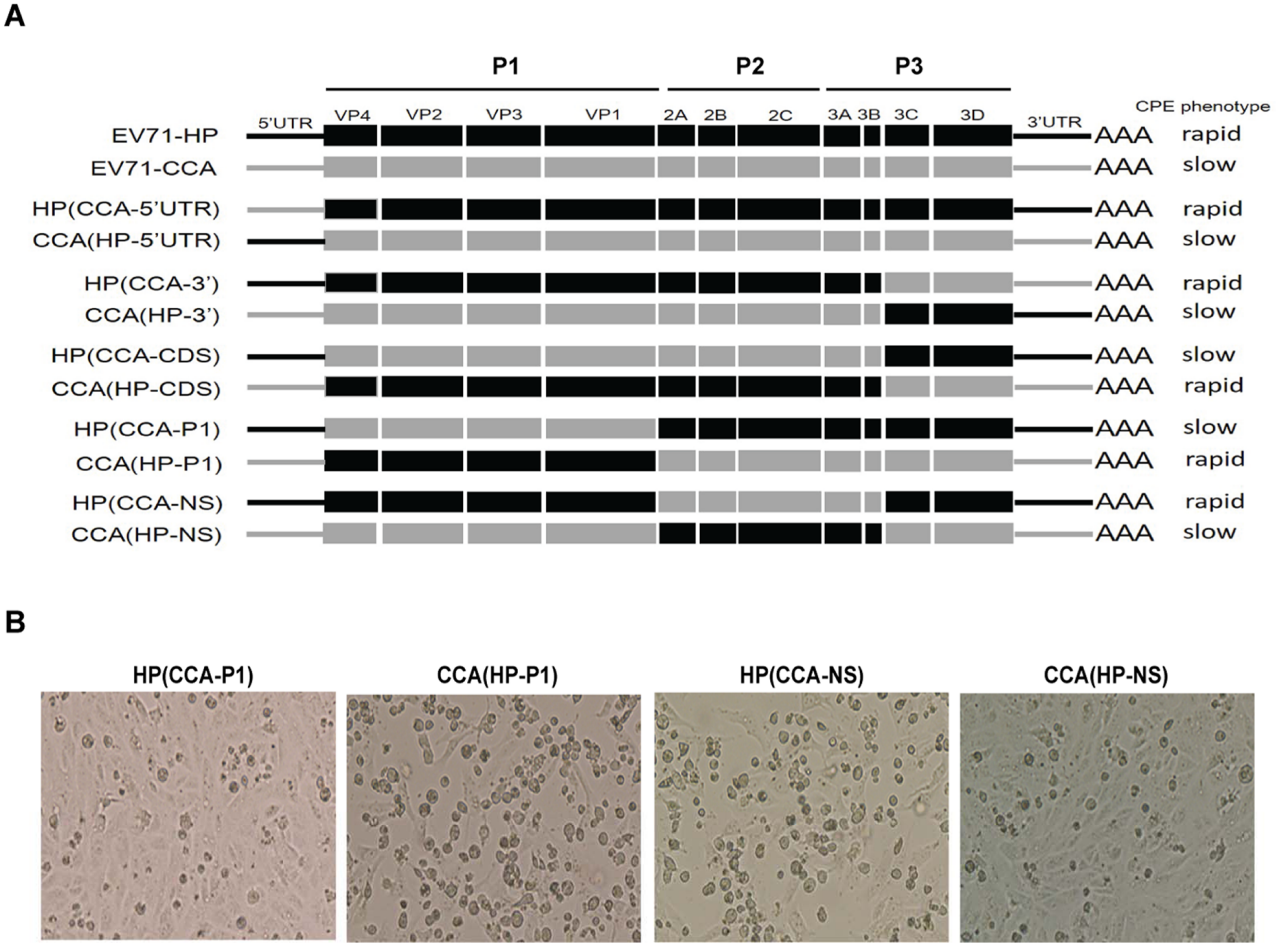
Growth phenotypes of chimeric recombinant viruses. (A) Schematic diagram of the chimeric recombinant constructs between EV71-HP and EV71-CCA. The growth phenotype of each chimeric virus is shown on the right side. (B) Cytopathic effects induced by the chimeric viruses. The CPE was recorded at 48 hours after viral genomic RNA transfection.

In order to determine the viral gene(s) that underline the distinct replication kinetics of the EV71-HP and EV71-CCA viruses, we swapped the genomic sequences between these two viruses and generated a panel of chimeric viruses (Fig 2A). Replication capacities of these viruses were assessed on the basis of the cytopathic effects and virus titers 48 hours after transfection of these viral RNA into Vero cells. EV71-HP was scored as the “Rapid type” relative to EV71-CCA that represented the “Slow type”, because much higher titer and faster formation of CPE were observed for EV71-HP. Among the chimeric viruses, exchange of the 5’UTR, the 3’ terminal region from 3C to 3’UTR region or the non-structural protein coding region (NS) from 2A to 3B region had no significant effect on the ability of parental strains to induce CPE, these include viruses HP(CCA-5’UTR)/CCA(HP-5’UTR), HP(CCA-3’)/CCA(HP-3’) and HP(CCA- NS)/CCA(HP-NS). However, exchange of the P1 region resulted in a complete switching of the infection phenotype of the parental viruses, as shown in Fig 2B that the CCA(HP-P1) virus exhibited a rapid growth phenotype whereas the HP(CCA-P1) virus became a slow one. Similar observation was made for viruses HP(CCA-CDS) and CCA(HP-CDS) that also had the P1 region swapped. These results indicate that the P1 region, which encodes viral proteins VP1 to VP4, determines the different growth phenotypes of EV71-HP and EV71-CCA.

### The A107 amino acid in VP1 is primarily responsible for the different replication kinetics of viruses EV71-HP and EV71-CCA

**Fig 3:**
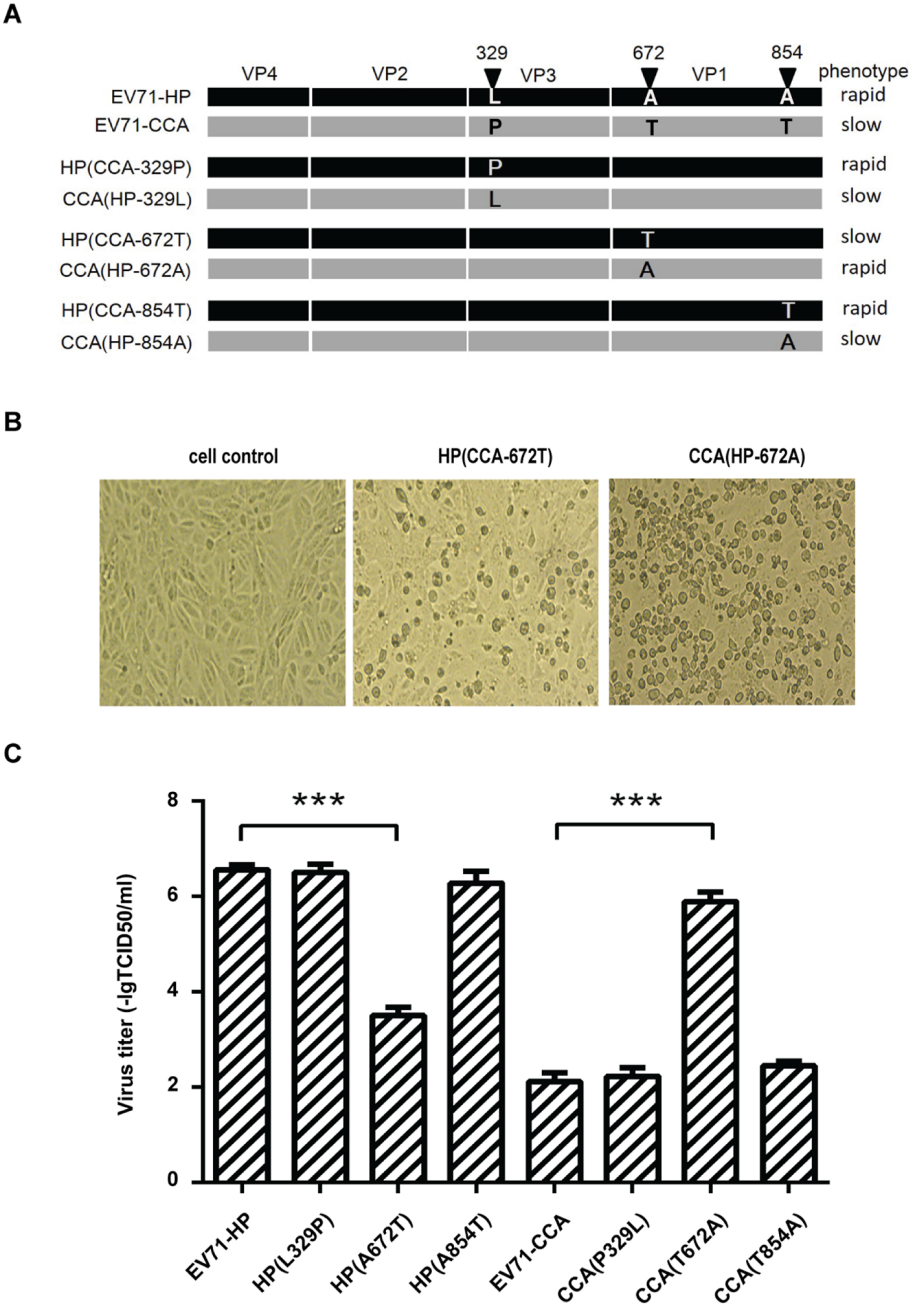
VP1^107^ is a genetic determinant of cell-culture growth phenotype of EV71. (A) Schematic illustration of the site-directed mutations in EV71-HP and EV71-CCA. The growth phenotype of each mutant is shown on the right side. (B) Cytopathic effect induced by the EV71 mutants. (C) Virus titers of the EV71 mutants. All assays were performed in triplicates. For each viral mutant, titers are means of three samples, error bars represent SEM. Three asterisks indicate P<0.01.

We next aligned the sequences of the P1 protein region between EV71-HP and EV71-CCA, and found three non-conservative amino acid mutations, including leucine (HP)/proline (CCA) at position 329 in capsid protein VP3, alanine (HP)/threonine (CCA) at positions 672 and 854 in capsid protein VP1 (Fig 3A). In order to determine which of these three mutations have impaired the replication of EV71-CCA, we swapped these amino acid residues in EV71-HP and EV71-CCA by site-directed mutagenesis (Fig 3A) and generated 6 recombinant viruses. Replication of these viruses was examined by recording CPE and determining virus titers 48 hours post transfection of viral RNA into Vero cells. The results showed that mutating the alanine residue at position 672 to threonine in EV71-HP (named HP(A672T)) dramatically delayed viral replication and caused a great reduction in virus titer (3.50 –lgTCID_50_/ml, P<0.01). Conversely, changing the T672 to A672 in EV71-CCA increased viral titer from the original value of 2.00 –lgTCID_50_/ml to 6.00 –lgTCID_50_/ml (P<0.01), a level observed for the EV71-HP virus (Fig 3B and 3C). In contrast, mutating the amino acids at position 329 or 854 did not affect virus titer (Fig 3C). These results demonstrate that the amino acid substitution at position 672 of the polyprotein (position 107 in the VP1 capsid protein, VP1^A107^) has undermined the replication of EV71-CCA.

### The role of the VP1^107^ residue in viral replication

**Fig 4:**
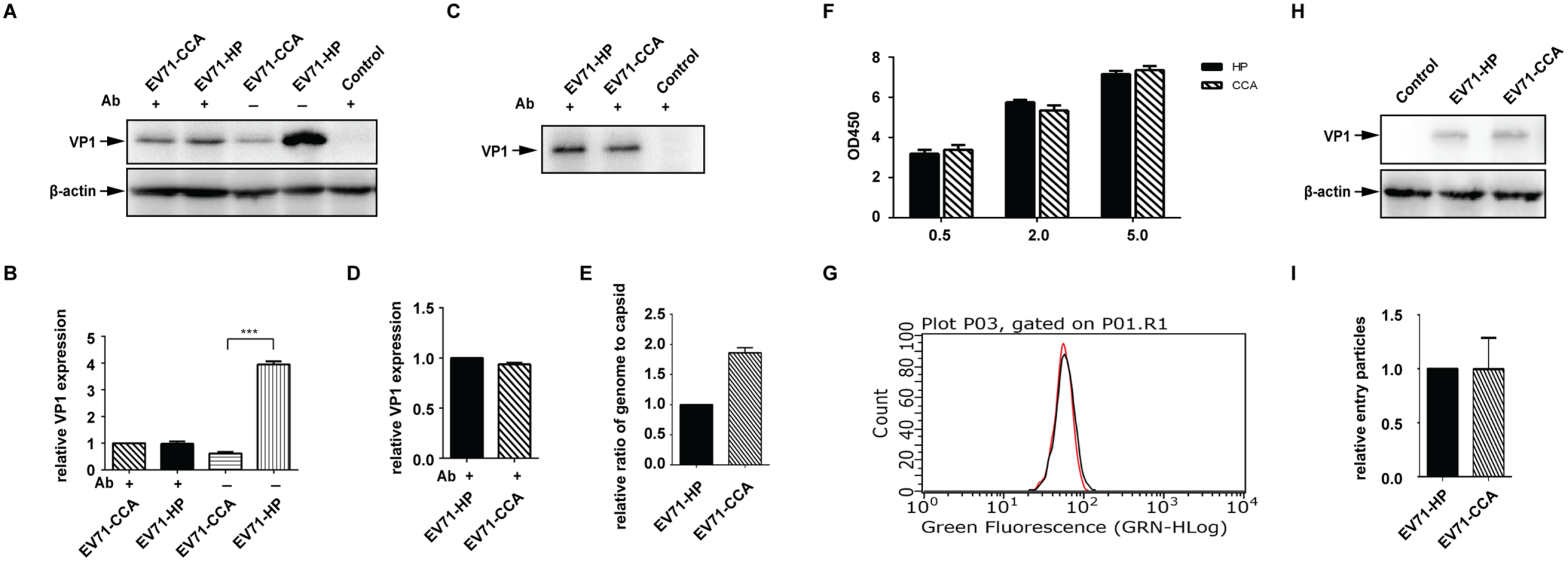
The VP1^107^ residue modulates viral spread in cell culture. Equal amounts of infectious RNA of EV71-HP and EV71-CCA were transfected into Vero cells, then treated with EV71 neutralizing antibody 2 hours post transfection. (A) Expression of viral protein in cells treated with the EV71 neutralizing antibody. Cell lysates were subjected to western blot analysis using the antibody specific for EV71 VP1. (B) The band density of each lane shown in panel A was quantified with ImageJ and expressed as fold increase relative to that of EV71-CCA that was treated with the antibody. β-actin was included as the internal control. Three asterisks indicate P<0.01. (C) Viral production from cells treated with the neutralizing antibody. Viral particles in supernatants were collected and purified by ultracentrifugation through a sucrose cushion, and analyzed in western blotting. (D) The band density in panel C was determined with ImageJ and expressed as fold increase relative to band density of EV71-HP. (E) Effect of VP1^107^ substitution on viral RNA packaging efficiency. The genomic RNA copies were determined by real time PCR, the amounts of capsid protein were determined by Bradford assay. The efficiency of viral RNA packaging is calculated as the ratio of RNA copies to capsid protein. The ratio of EV71-CCA was expressed as fold increase relative to that of EV71-HP. (F) Effect of VP1^107^ substitution on virus attachment as measured by cell-ELISA. Vero cells were inoculated with virus at different MOIs. The attached viruses were stained withEV71 VP1 antibody and HRP-conjugated secondary antibody. TMB peroxidase substrate was added and the absorbance at OD 450 nm was measured. (G) Effect of VP1^107^ substitution on virus attachment as measured by flow cytometry. The stained cells shown in panel A were detected by flow cytometer. The red and black curves indicate cells that were infected with EV71-HP and EV71-CCA, respectively. (H) Effect of VP1^107^ substitution on virus internalization. Vero cells were inoculated with equal amounts of EV71-HP or EV71-CCA. After synchronized adsorption at 4°C for 1 hour, the unbound virions were removed by PBS wash. Virus internalization was performed by incubation at 37°C for 30 minutes. Subtilisin A was used to remove the non-internalized virions. The internalized virions were detected by western blotting. (I) The band density of VP1 of internalized virions shown in panel H was quantified with ImageJ, and expressed as fold increase relative to that of EV71-HP.

We next asked which stage of EV71 replication is affected by the A107T mutation in VP1. To this end, equal amounts of full-length RNA of EV71-HP and EV71-CCA were transfected into Vero cells. The neutralizing antibody was then added into cell culture in order to block a second round of viral infection and thus stop viral spread. Viral replication was monitored by measuring the capsid protein VP1 in Western blot (Fig 4A). Similar levels of VP1 were observed for both EV71-HP and EV71-CCA in the presence of neutralizing antibody, whereas in the absence of neutralizing antibody transfection of the EV71-HP RNA led to the expression of more than 4-fold more VP1 than that by EV71-CCA (Fig 4B). We also collected and purified viral particles in the equal volumes of supernatants from the virus-producing cells that had been treated with neutralizing antibody, and determined the amounts of viral particles by Western blot using antibody specific for VP1 (Fig 4C). Similar levels of EV71-HP and EV71-CCA particles were observed (Fig 4D). These data suggest that the A107T mutation in VP1 does not affect viral gene expression, or the levels of virus particle production, but rather impairs the infection of new target cells by the progeny EV71-CCA particles, thus suppressing virus spread.

In order to identify the defect(s) that have led to the impaired infectivity of the EV71-CCA particles, we first quantified viral genomic RNA and the amount of capsid protein in the EV71-HP and EV71-CCA virions (S2 Fig). Interestingly, the ratio of genome RNA copies to capsid protein in EV71-CCA was higher than that of EV71-HP (Fig 4E).

VP1 capsid protein plays a central role in the attachment of EV71 virions to host receptors [16, 24]. We thus measured the cell attachment of EV71-HP and EV71-CCA using a cell-ELISA as described previously [25]. Similar amounts of both viruses were found bound to the cell surface using different MOI of viruses, suggesting no significant difference in cell attachment (Fig 4F). This observation was further verified by the data of flow cytometry. The signals of EV71-HP (in red line) and EV71-CCA (in black line) overlapped completely (Fig 4G), showing comparable amounts of cell attached virions for both viruses.

Next, we further investigated whether the EV71-HP and EV71-CCA viruses were internalized by cells at different rates. Vero cells were inoculated with equal amounts of these two viruses. Following synchronized attachment to cells at 4°C and cell internalization at 37°C, virions that did not enter the cells were removed by digestion with subtilisin. VP1 protein of the internalized virions was analyzed by Western blot (Fig 4H). The results showed similar levels of internalized VP1 protein for both viruses (Fig 4I), indicating that the cell internalization of EV71-CCA is not impaired compared to EV71-HP. Taken together, these results suggest that the A107T mutation in VP1 affects a step of viral entry after cell internalization of virus particles.

### Uncoating of EV71-CCA is delayed compared to EV71-HP

**Fig 5:**
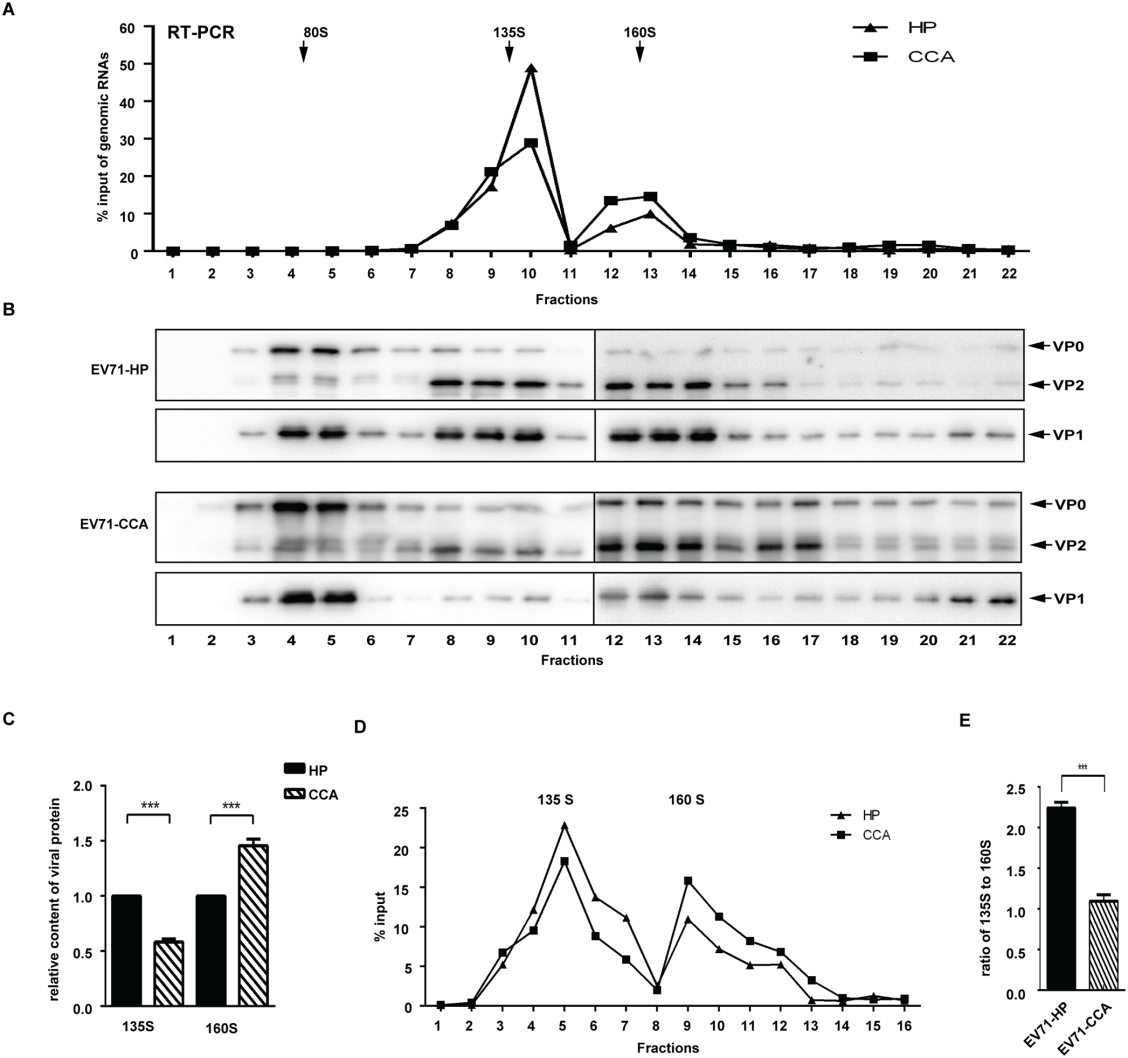
EV71-CCA uncoating is impaired. EV71-HP and EV71-CCA particles were analyzed in a 15%-35% discontinuous sucrose gradient ultracentrifugation. (A) Viral genome RNA in each fraction was quantified by RT-PCR. The data were normalized to input genome RNA (total copy number of genomic RNA in all fractions) (B) Western blotting of each fraction using anti-EV71 VP0/VP2 and VP1 antibody, respectively. (C) Relative content of viral protein in the 135S virions and in the 160S virions. The band density of VP2 protein was determined with ImageJ, the relative amount of EV71-CCA was expressed as fold increase relative to EV71-HP. (D) Gradients were fractionated in small volume of aliquots (0.25 ml), then genomic RNA in each fractions containing 135S and 160S particles were quantified. (E) Uncoating efficiency of EV71. The area under the curve in (D) represents the amount of viral particles. The ratio of the amount of 135S particles to the 160S particles was calculated to represent uncoating efficiency of EV71. Three asterisks indicate P<0.01.

EV71 virion uncoating after its entry is a prerequisite for productive infection, enabling the release of viral RNA genome into the cytoplasm. EV71 uncoating involves conformational changes in capsid, generating 3 types of virus particles: the mature virions sedimentated at 160S, the A-particles sedimentated at 135S and the empty viral particles sedimentated at 80S [18]. The first step of this dynamic process is conversion of the mature virion (160S) into an expanded intermediate or A-particle (135S), which is characterized by the release of a pocket factor to open the two-fold symmetry axis channels [15, 17]. Then, the A-particle is converted into the empty capsid (80S), which is accompanied by the release of the RNA genome into the cytoplasm. To compare the uncoating of EV71-HP and EV71-CCA, we first separated the mature virions and the A-particles of both strains by ultracentrifugation through a 15%-35% discontinuous sucrose gradient. For each fraction, the virion-associated genomic RNA was determined by RT-PCR (Fig 5A) and the pelleted capsid protein by Western blot (Fig 5B). Results of both experiments revealed two peaks of RNA-containing virions in fractions 8 to 10 (135S) and fractions 12 to 14 (160S), which correspond to the A-particles and mature virions, respectively. Notably, in 160S fractions, the amounts of both virion-associated genome RNA and viral protein of EV71-HP were less than those of EV71-CCA, while a reverse trend was found in 135S fractions (Fig 5A and 5C). This suggests a defect in uncoating for EV71-CCA compared with EV71-HP, which is reflected in the accumulated 160S particles of EV71-CCA. For further detailed comparison between both strains in uncoating efficiency, gradients were fractionated in small volume of aliquots (0.25 ml), then genomic RNA in both 135S and 160S particles were quantified and used to determine amount of different forms of particles (Fig 5D). The area under the curve (AUC) represents the amount of virus particles. The ratio of the amount of 135S particles to the 160S particles was used to assess the uncoating efficiency of EV71. The results showed an approximate 2-fold decrease in this ratio for EV71-CCA compared with EV71-HP (Fig 5E), suggesting that the uncoating efficiency of the EV71-HP strain is significantly higher than that of the EV71-CCA strain (P<0.01).

### The A107 residue in VP1 regulates the cleavage of precursor VP0 and virion maturation

**Fig 6:**
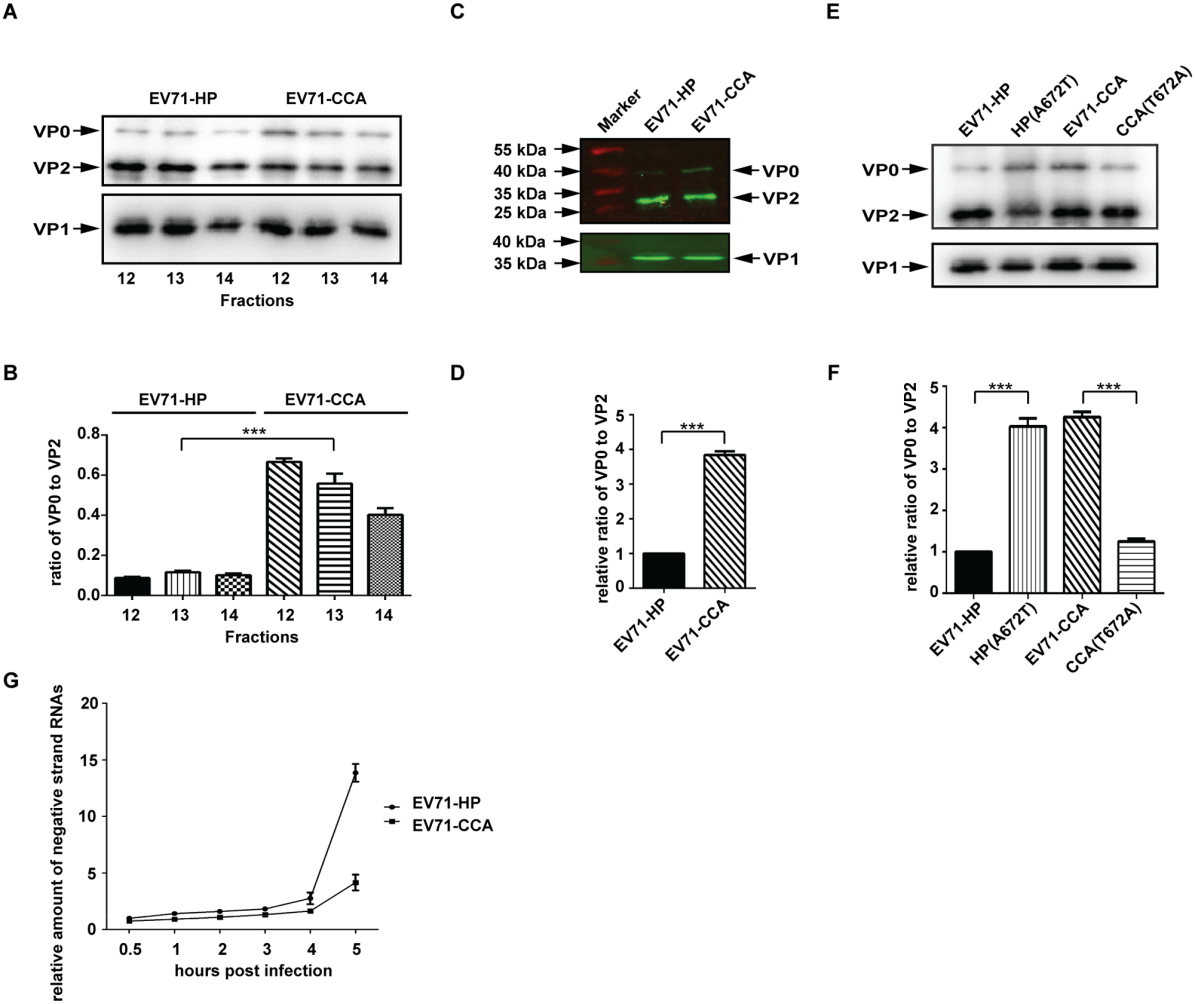
The VP1^107^ residue regulates the cleavage of precursor VP0. (A) Protein composition of the 160S particles (fractions of 12–14) of EV71-HP and EV71-CCA. The corresponding fractions of discontinuous sucrose density gradient were concentrated by ultracentrifugation and then subjected to western blotting with anti-EV71 VP0/VP2 and VP1 antibody, respectively. (B) VP0 cleavage efficiency of EV71. The band density of VP0 and VP2 in (A) were quantified using ImageJ, and the data were used to calculate the ratio of VP0 to VP2, which represents the VP0 cleavage efficiency. (C) Western blotting analysis of the 160S particles of EV71 by the Odyssey infrared imaging system. (D) VP0 cleavage efficiency of EV71. The band density of VP0 and VP2 protein shown in (C) was quantified and used to determine VP0 cleavage efficiency. (E) Western blotting analysis of 160S particles of EV71-HP, HP(A672T), CCA and CCA(T672A). (F) VP0 cleavage efficiency of the above substitutional mutants. Three asterisks indicate P<0.01. (G) RNA replication of CCA strain is delayed relative to HP strain. The cells were inoculated with equal amount of HP strain or CCA strain. After synchronized viral adsorption, the infected cells were incubated at 37°C and collected by Trizol reagent at indicated time points post infection. The negative strand genomic RNA of each sample was relatively quantified by RT-PCR and expressed as fold increase relative to that of sample collected at 0.5 hour post infection.

We noted a considerably higher amount of VP0 protein in the mature virions of the EV71-CCA strain compared to the EV71-HP strain that were sedimentated to fractions 12 to 14 (160S) (Fig 5B). The precursor VP0 protein is primarily associated with the procapsids and provirions, and is processed into VP2 and VP4 during viral assembly and maturation, an auto-catalysis process as a result of the interactions between the viral structural proteins and viral RNA genome [13]. The accumulation of VP0 in the mature virions indicates incomplete VP0 cleavage and impaired virion maturation. To further examine the difference in VP0 cleavage efficiency between the EV71-HP and the EV71-CCA strain, fractions 12 to 14 containing the mature virions (160S) for both strains were analyzed in the same Western blot using the antibody recognizing both VP0 and VP2 (Fig 6A). The amounts of VP0 and VP2 were determined and used to calculate the ratio of VP0 vs VP2 proteins, the values reflect the relative amount of VP0 in the mature virions (Fig 6B). The results in Fig 6B show that the relative amount of VP0 in the 160S fractions of the EV71-CCA strain is 6-fold higher than that of the EV71-HP strain. We further verified the significant increase in VP0 in the mature EV71-CCA particles by protein quantitative analysis using the Odyssey infrared imaging system (Fig 6C and 6D). To exclude the possibility that the antigenicity of the commercial VP0 antibody used is different against the VP0/VP2 from CCA strain and HP strain, we have visualized viral protein in 160S fractions of HP and CCA strains with a silver staining method (S3 Fig). The result shows significantly increased amount of VP0 as well as VP0/VP2 ratio in CCA strain than HP strain. In agreement with western blot analysis, this suggests an inefficient VP0 cleavage in CCA strain. These results collectively suggest that the impairment in VP0 cleavage and virion maturation occurs during assembly of the EV71-CCA strain. Since the mature VP4 protein is expelled from viral capsid during the formation of the A-particle [19], we propose that the impaired maturation of EV71 virions as a result of the inefficient cleavage of VP0 into VP2 and VP4 hinders the conversion of mature particles (160S) to A-particles (135S) during viral uncoating, ultimately diminishing viral infectivity.

To further demonstrate that the defect in VP0 cleavage in EV71-CCA is caused by the A107T mutation in VP1, we examined the cleavage of VP0 in viruses CCA (T672A) and HP (A672T), both viruses have shown remarkable change in viral infectivity by a single amino acid substitution at VP1^107^ compared to their parental strains (Fig 3). These two mutated viruses and their parental strains were separated in a 15%-35% discontinuous sucrose gradient to collect the 160S particles, which were examined in Western blot (Fig 6E). The quantitative analysis of western blot data revealed a significant increase in the ratio of VP0 to VP2 in HP(A672T) compared with its parental strain, which contrasts a remarkable decrease in the ratio for CCA(T672A) (Fig 6F). This correlation between viral infectivity and the cleavage of VP0 suggests that the VP1^A107^ residue contributes to viral infectivity by regulating the cleavage of VP0 and subsequent viral uncoating.

EV71 virion uncoating is a prerequisite for enabling the release of viral RNA genome into the cytoplasm. Due to the insufficient VP0 cleavage, the inefficient uncoating of the EV71-CCA strain shall result in delayed genomic RNA release and consequently RNA replication. In agreement with the hypothesis, we found that RNA replication in EV71-CCA was delayed in the early stage of the infection compared with that of EV71-HP (Fig 6G). This provide further evidence supporting the defect of EV71-CCA in viral uncoating process.

### Molecular dynamic (MD) simulation of VP1^A107^ substitution

**Fig 7:**
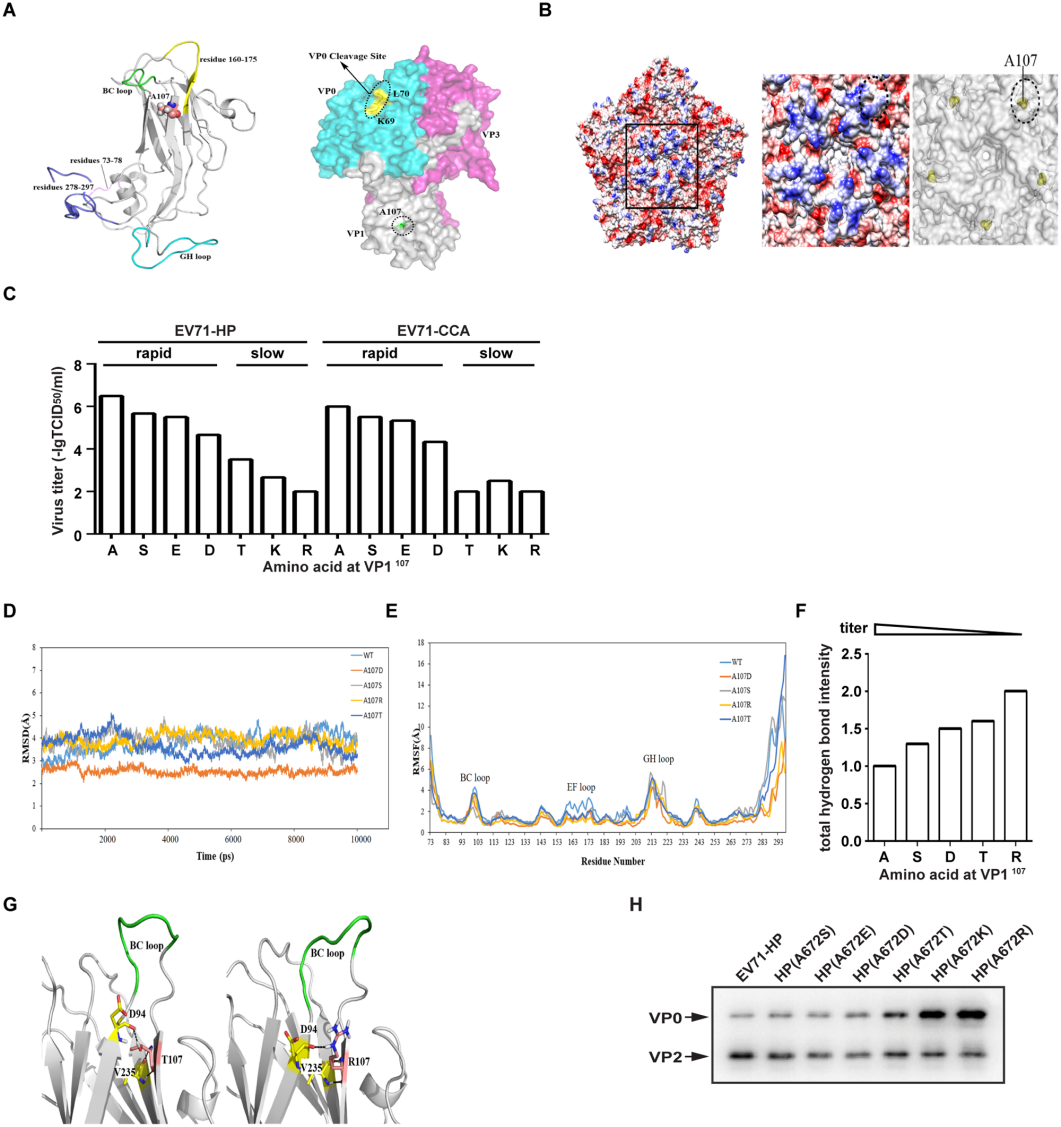
Structural analysis of substitutions at VP1^107^. (A)Left panel: Ribon structure of the equilibrated VP1 from the EV71 particle (PDB ID 3VBU). BC loop is denoted in green, EF loop in yellow, residue VP1-107A is lined out as cartoon atoms. Right panel: Crystal structure of empty human Enterovirus 71 particle (PDB ID 3VBU). The molecule is rendered as a surface that is colored by chain. Here the color code is that blue is VP0, white is VP1, and magenta is VP3. Dotted ovals indicate VP1 A107, which is shown in green spheres. The VP0 cleavage site between K69 residue and L70 residue is colored in yellow. (B) Overall structure of the EV71 virion. In the left panel, electrostatic surface is colored to indicate the five icosahedral symmetric units. Negatively charged surfaces are shown in red, positively charged areas in blue. Molecular surfaces of black-boxed area around the five-fold axis of symmetry are enlarged and shown in the middle panel. Dotted ovals indicate a beta-sheet (residue 106–109). The right panel shows the position of VP1^107^ in yellow, surrounding the five-fold axis of symmetry. (C) Infectious titers of substitutional mutants for EV71-HP and EV71-CCA. The growth phenotype of each mutant is shown above. (D) The root mean square deviation (RMSD) values of the backbone Cα atoms of each substitutional mutant. (E) Root mean square fluctuation (RMSF) values of each substitution mutant. The BC loop, EF loop and GH loop are indicated. (F) Analysis of the VP1^107^ residue associated hydrogen-bond networks. The intensity of hydrogen bond is visualized as the number calculated by occupancy of hydrogen bond with weighting coefficient. The infectious titer of each substitutional mutant has also been shown. (G) Snapshots obtained from 10ns trajectories for the A107T (left) and the A107R (right) mutants that show a slow growth phenotype. The BC loop (residues 96–102) is colored in green, together with the residue D94 and V235 in yellow. For clarity, residue 107 has been colored in salmon. (H) Western blotting analysis of 160S particles of EV71-HP mutations using anti-EV71 VP0/VP2 antibody.

Recent crystal structures [15] of the empty EV71 virion illustrate that VP1^A107^ is located on the βC of VP1 (Fig 7A), one of the two β-sheets linked by BC loop, flattened away from the five-fold axis of symmetry (Fig 7B). To determine the level of conservation of the VP1^A107^ in EV71 isolates, we analyzed the amino acid sequences of VP1 βC that are available in GenBank database (as of January 2016, 3777 sequences). VP1-107A is found in virtually all the available sequences of VP1 βC. This high conservation of VP1^A107^ indicates its important role in EV71 replication.

To further investigate the function of the VP1^A107^ residue, we substituted this amino acid for other residues with different electrostatic potential and the side chain in both EV71-HP and EV71-CCA strain and examined the replication of these viral mutants. The results in Fig 7C show that among all the amino acids tested, A107 gave rise to the highest viral titers for both the EV71-HP and the EV71-CCA strain, the positively charged residues K and R were much less well tolerated at this position by the virus compared to negatively charged E and D, which explains the high conservation of the alanine amino acid at position 107 in VP1.

We next investigated the structural mechanism underlying the replication ability of the above VP1 mutated viruses through MD simulations of EV71-HP (A107) and the A107T, A107R, A107D and A107S mutants. In the analysis, the root mean square deviation (RMSD) values of the backbone Cα atoms were calculated and plotted in Fig 7D. Root-mean-square fluctuation (RMSF) values provide details on fluctuation of each residue over simulation time, reflecting the effect of different substitutions on the structural stability of the region tested [26]. As shown in Fig 7E, the terminal regions (residues 73–78 and 278–297), the BC loop, and the GH loop exhibited very high RMSF values in all strains. Notably, the RMSF values of residues 160–175 of the EF loop (highlighted in yellow in Fig 7A) for the EV71-HP strain (cyan plot, Fig 7E) are greater than those of other mutants, indicating that more unstable conformations were adopted by this loop in the EV71-HP strain. We therefore propose that a flexible conformation of the EF loop or the surrounding region contributes to efficient maturation and replication of EV71 virions.

### Analysis of hydrogen-bond networks associated with the VP1^A107^ residue

In addition to viral infectious titers, we also examined the growth phenotypes of mutant viruses in cultured cells by monitoring the induction of CPE. As shown in Fig 7C, all the strains that had higher than 4 of -lgTCID_50_/ml exhibited rapid growth phenotype, including A, S, E and D at VP1^107^ in both the EV71-HP and EV71-CCA strains. However, substitution of VP1^107^ for T, K and R resulted in a slow growth phenotype, and these mutants produced lower than 3 of -lgTCID_50_/ml. These data indicate that viral replication ability is well correlated with viral growth phenotype. Substitution of the VP1^107^ residue generated significant variations in viral infectious titers and viral growth phenotypes, which provides an opportunity to explore the structural basis for the replication ability of these various mutants. However, the RMSF analysis used above did not reveal much difference for most of substitutional mutants except for A107. As the hydrogen-bond interaction plays a crucial role for the structure and function of protein, we further investigated the hydrogen-bond networks associated with the VP1^107^ residue for each amino acid substitution. The results in Table 1 show that the backbone hydrogen bonds formed between residues 107 and V235 are conserved in EV71-HP and all the tested mutations of VP1, indicating their importance in the structural stability. Notably, the analysis data showed that the number and occupancy of hydrogen-bonds for these substitutions increased concurrent with decrease in viral replication. To better visualize the results, we added together all occupancy of hydrogen bonds between each pair of residues, and then classified them into three groups, including high, middle, low possibility of hydrogen bonding if total sum of occupancy is >100%, 100–50% and <50%, respectively. Next, we calculated the total number of hydrogen bonds for each substitution by setting the weighting coefficient of high, middle, low possibility of hydrogen bonding as 1, 0.5 and 0.1, respectively. This number is used to assess the stability of the region surrounding the VP1^107^ residue, and the data are summarized in Fig 7F, showing that the total number of hydrogen bonds for each substitution is inversely correlated to the infectious titers of the mutated viruses. This observation further supports the hypothesis that a flexible conformation of the region surrounding the VP1^107^ residue is required for efficient replication of EV71.

**Table 1:** Comparison of VP1^107^ associated hydrogen-bond networks for EV71-HP and four VP1 mutants.

VP1^107^ Substitution	H-bond	Donor	Acceptor	Distance(Å)	angle(°)	Occupied (%)
**WT**	A107–V235	V235-N-H	A107-O	2.86	160.26	79
		A107-N-H	V235-O	2.90	155.17	35
**A107S**	S107–V235	V235-N-H	S107-O	2.86	160.82	81
		S107-N-H	V235-O	2.88	158.98	71
	S107–N102	N102-ND2-HD22	S107-OG	2.89	158.84	28
	S107–Y106	S107-OG-HG	Y106-O	2.80	153.94	12
	S107–N108	N108-N-H	S107-OG	2.88	127.42	5
**A107D**	D107–V235	V235-N-H	D107-O	2.87	164.51	74
		D107-N-H	V235-O	2.89	158.74	49
	D107–N108	N108-N-H	D107-OD2	2.84	147.35	34
		N108-N-H	D107-OD1	2.83	147.18	31
**A107T**	T107–D94	T107-OG1-HG1	D94-O	2.78	157.66	71
	T107–V235	T107-N-H	V235-O	2.91	159.79	34
		V235-N-H	T107-O	2.86	160.89	81
	T107-N108	N108-N-H	T107-OG1	2.89	124.85	7
**A107R**	R107–D94	R107-NE-HE	D94-O	2.82	156.13	53
		R107-NH1-HH11	D94-O	2.80	150.44	22
		R107-NH2-HH21	D94-OD2	2.79	156.74	18
		R107-NH2-HH21	D94-OD1	2.80	153.39	14
		R107-NH1-HH12	D94-OD2	2.82	139.92	6
	R107–V235	R107-N-H	V235-O	2.89	156.97	50
		V235-N-H	R107-O	2.87	162.5	73

Only hydrogen bonds that are resident for more than 5% of the simulation are shown.

Interestingly, D94 is involved in hydrogen-bonding only in the two mutants that exhibited slow growth phenotype. For A107T, the side-chain hydroxyl group of T107 forms strong H-bond with the main chain oxygen atom of D94 with the percentage occupancy of 71% (Fig 7G, left). For A107R, both the main chain and side chain oxygen atoms of D94 form hydrogen bonds with the guanidinium moiety of R107 (Fig 7G, right). When the main chain H-bond disappears, the side chain one still present. Therefore, the H-bond probability between D94 and R107 is inferred higher than 76%. Those hydrogen bonds are typically present in the middle of the trajectory. In contrast, we did not observe any H-bond interactions involving D94 for VP1 mutants that exhibit rapid growth phenotype. When mutated to aspartic acid, the carboxylate oxygen atoms of D107 formed H-bonds with the backbone N-H of N108. Although S107 interacted with the Y106, N108 and N102 residues adjacent to it, the percentage occupancy of these H-bonds in trajectories is very low. These results suggest that the H-bonds involving residue D94 for the A107T and A107R mutants significantly enhance the stability of the two β-sheets linked by the BC loop, resulting in impaired replication ability and slow viral growth.

Finally, we examined the VP0 cleavage of these mutants tested above. As shown in Fig 7H, the VP0 content is increased accompanied with decreased viral replication ability. This provides biochemical evidence supporting the conclusion from MD simulation and hydrogen-bond networks analysis, and the hypothesis that a flexible conformation of the region surrounding the VP1^107^ residue is required for efficient VP0 cleavage and virion maturation of EV71.

## Discussion

A number of virulence determinants for EV71 have been reported, which may influence virus assembly, attachment, replication, or cell tropism. In this study, we have identified a single amino acid A107 in VP1 as the genetic determinant of viral virulence that functions through regulating viral maturation. This A107 in VP1 is conserved in the 3777 EV71 strains that are available in the GenBank database. This high conservation of VP1^A107^, regardless of different EV71 lineages, indicates the important function of this residue in EV71 replication.

Our mechanistic studies showed that mutation of VP1^A107^ impairs the cleavage of capsid precursor protein VP0, which occurs at the final stage of enterovirus assembly, involving an inward radial collapse of the provirion to yield the mature virus particle. Infectivity analysis showed that a single amino acid substitution (A to T) at VP1^107^ caused slow viral growth and dramatically reduced virus titer (3.50 –lgTCID_50_/ml), concurrent with impaired VP0 cleavage. This correlation between diminished viral infectivity and impaired cleavage of VP0 among the substitutions of the VP1^107^ residue further strengthens the important role of VP0 cleavage in viral infectivity [27].

Cleavage of VP0 has also been reported being critical for the infectivity of other picornaviruses by regulating the assembly of virions and stability of mature particles. Impaired cleavage of VP0 in Poliovirus causes accumulation of the assembly intermediates, i.e., empty-capsid-like structures [13] and provirions [12], while some substitution mutations at VP2^195^ result in the assembly of a highly unstable 150S virus particle resembling mature virions except carrying the uncleaved VP0 [28]. Herein, we found that the defective VP0 cleavage in the EV71-CCA strain hinders the conformational conversion from the 160S virions to the 135S A-particles, suggesting an important role of VP0 cleavage in EV71 uncoating. One model for picornavirus uncoating describes that binding of viral receptor triggers conformational changes of the capsid proteins, leading to the formation of the expanded A-particle with a sediment value of 135S [18]. During the expansion process, VP4 is expelled from the capsid, allowing the formation of a gateway to release viral genome [17]. Therefore, the failure to dissociate VP4 from the 160S virions, due to inefficient cleavage of VP0, may impair the formation of A-particles in the VP1^107^ mutated virus. The defective uncoating consequently prevents release of genomic RNA, resulting in reduction in viral replication. In agreement with this, we found that RNA replication in EV71-CCA was delayed in the early stage of the infection compared with that of EV71-HP (Fig 6G).

Currently, the mechanism underlying the cleavage of VP0 in EV71 is largely unknown. Based on the study of Poliovirus, two conserved amino acids, VP2-195H and VP2-194P, in the immediate vicinity of the scissile bond as well as two bound water molecules execute the maturation cleavage [13]. The imidazole ring of VP2-195H is placed in an appropriate spatial configuration to activate the local water molecules due to a tight bend in the main chain formed by VP2-194P, which leads to a nucleophilic attack of the scissile bond to catalyze the hydrolysis of the peptide bond. The cleavage site in VP0 is located at the rim of a large trefoil-shaped hydrophobic depression centered on the particle three-fold axis in the inner surface of the empty capsid [13]. However, the residue VP1^107^ is located on the C β-strand of VP1, adjacent to the end of the BC loop, which is mostly exposed on the capsid surface at a canyon-like depression surrounding the five-fold axis of symmetry (Fig 7B). In view of the distance of the VP1^107^ residue from the VP0 cleavage site, the residue VP1^107^ may regulate VP0 cleavage and viral maturation in an indirect manner, possibly by conformational alteration of the virion precursor rather than by directly interfering with the active site of proteolysis.

The efficiency of VP0 cleavage is dependent on the folding conformation of the peptide chains surrounding the scissile bond [13, 28]. The VP2-194P mutants carrying serine or glycine substitutions cleave VP0 inefficiently, which may result from configurational alteration of VP2-195H caused by higher degrees of rotational freedom of the main chain [28]. In addition to this, configuration of the local residues away from the VP0 cleavage site may also have a dramatic effect on the cleavage event. Previous studies with hepatitis A virus, rhinovirus, and poliovirus have shown that a series of conformational changes and intra-subunit communications within the particle occur during the maturation cleavage of VP0 [29]. For example, some mutations at VP2^76^, distal to the scissile bond of VP0, prevent the maturation cleavage in poliovirus, suggesting an involvement of conformational changes around the cleavage site [30].

Analysis of associated hydrogen-bond networks showed a trend that the increasing number and occupancy of hydrogen-bond for VP1^107^ correlate with decreased viral replication. More specifically, the D94 residue in the B β-strand of VP1, adjacent to the other end of BC loop, was predicted to form hydrogen-bonds with the VP1^107^ residue in viruses that exhibit slow growth phenotype (Fig 7G). This suggests that sufficient flexibility of the VP1 BC loop or the region surrounding VP1^107^ residue, which results from less hydrogen-bonding, might favor VP0 conformational changes that are required for the efficient cleavage of VP0, therefore facilitating viral uncoating and viral replication. One possible interesting avenue of this finding might be providing methods for virus vaccine stabilization; similar approaches have shown some promise in the design of synthetic FMDV vaccines [31]. Increasing stability of the capsid by introducing substitution mutation at VP1^107^ may prevent the dissociation of intact virus particles and subsequently the loss in immunogenicity of vaccine. However, this would need to be demonstrated experimentally in the future.

Although much effort has been made to discover molecular determinants for EV71 virulence, the relevant knowledge is still incomplete. One critical domain in VP1 protein near the 5-fold vertices of the EV71 capsid has been reported as an important contributor to viral virulence. This region is critical for receptor binding and necessary for the stability of infectious virions. Single mutations (G145E, E145Q, or K244E) in the conserved, positively charged 5-fold region of the VP1 capsid protein were identified as important sites in EV71 for mouse adaptation and neuropathogenesis in cynomolgus monkey [32–34]. Further studies revealed that K244E was necessary for increased virulence and neurotropism of mouse adapted EV71 (mEV71) in adult interferon deficient mice. Meanwhile, another VP1 mutation (H37R) was required for mEV71 (K244E) recovery in rhabdomyosarcoma (RD) cells [35]. The VP1-145 residue controls PSGL-1 binding by modulating the exposure of VP1-244K, which determines cell tropism [24]. A canyon region around residue VP1-172Q has also been shown to interact with the variable region on SCARB2, another main host receptor of EV71 [16]. Moreover, substitutions in other regions of VP1 protein were shown being important for EV71 virulence. The substitution L97R within the VP1 BC loop confers a replicative advantage in SH-SY5Y cells of neuroblastoma origin with a frequent association of a second non-conservative mutation (E167G or E167A) in the VP1 EF loop [36].

In summary, we have demonstrated that VP1^107A^ is critical for EV71 replication, and is highly conserved among all viral genogroups. The results of mechanistic study suggest that the VP1^107^ residue modulates the flexibility of the VP1 BC loop, regulates VP0 cleavage and subsequent viral uncoating. Characterization of this novel determinant of EV71 virulence may provide new opportunities for vaccine development and drug discovery.

## Materials and methods

### Cells and viruses

African green monkey kidney (Vero) cells (ATCC CCL-81) and human rhabdomyosarcoma (RD) cells (ATCC CCL-136) were maintained in Dulbecco’s modified Eagle’s medium. EV71 strain HP (EV71-HP) was isolated from the patient with the nervous system diseases in Fuyang outbreak during 2008. EV71 strain CCA (EV71-CCA) was a cell adapted virus that were generated by series passage *in vitro*. Both strains were plaque-purified in Vero cells. Isolated plaques were passaged on RD cells to increase the titer for use in subsequent assays.

### RNA extraction, cDNA synthesis and nucleotide sequence analysis

Viral RNA was extracted from the culture medium of infected cells with Trizol LS reagent according to the manufacturer’s instructions. cDNA synthesis was performed by reverse transcription PCR as described previously [37].

### Construction of full-length infectious cDNA clones and mutants of EV71-HP and EV71-CCA

The full-length cDNA clones of EV71-HP (pEV71-HP) and EV71-CCA (pEV71-CCA) were constructed as described previously [38]. The complete genome sequences of the two EV71 strains were submitted to the GenBank database under accession numbers KY074643 and KY074644. A diagram of the chimeric recombinant cDNA clones is presented in Fig 2. All recombinant viruses were constructed by recombination technology using the plasmids pEV71-HP and pEV71-CCA as the templates. Names of the recombinant constructs are presented in the form of pA(B-C), where A is the backbone virus, B is the donor virus, and C is the exchanged genomic region.

cDNA clones of various point mutations were prepared by site-directed mutagenesis via PCR with specific pairs of primers using the infectious clone of pEV71-HP or pEV71-CCA as the template. The sequences of the primers used for site directed mutagenesis are as follows.

FY-672(S)+: CTAACCCAAATGGTTATTCCAACTGGGACATAGAC;

FY-672(S)-: GTCTATGTCCCAGTTGGAATAACCATTTGGGTTAG;

FY-672(E)+: CTAACCCAAATGGTTATGAGAACTGGGACATAGACATA;

FY-672(E)-: TATGTCTATGTCCCAGTTCTCATAACCATTTGGGTTAG;

FY-672(D)+: CTAACCCAAATGGTTATGACAACTGGGACATAGACAT;

FY-672(D)-: ATGTCTATGTCCCAGTTGTCATAACCATTTGGGTTAG;

FY-672(T)+: CTAACCCAAATGGTTATACCAACTGGGACATAGAC;

FY-672(T)-: GTCTATGTCCCAGTTGGTATAACCATTTGGGTTAG;

FY-672(K)+: CTAACCCAAATGGTTATAAGAACTGGGACATAGACATA;

FY-672(K)-: TATGTCTATGTCCCAGTTCTTATAACCATTTGGGTTAG;

FY-672(R)+: CTAACCCAAATGGTTATAGGAACTGGGACATAGACATA;

FY-672(R)-: TATGTCTATGTCCCAGTTCCTATAACCATTTGGGTTAG;

ZD-672(A)+: CTAACCCAAATGGTTATGCCAACTGGGACATAGAT;

ZD-672(A)-: ATCTATGTCCCAGTTGGCATAACCATTTGGGTTAG;

ZD-672(S)+: CTAACCCAAATGGTTATTCCAACTGGGACATAGAT;

ZD-672(S)-: ATCTATGTCCCAGTTGGAATAACCATTTGGGTTAG;

ZD-672(E)+: CTAACCCAAATGGTTATGAGAACTGGGACATAGATATA;

ZD-672(E)-: TATATCTATGTCCCAGTTCTCATAACCATTTGGGTTAG;

ZD-672(D)+: CTAACCCAAATGGTTATGACAACTGGGACATAGATAT;

ZD-672(D)-: ATATCTATGTCCCAGTTGTCATAACCATTTGGGTTAG;

ZD-672(K)+: CTAACCCAAATGGTTATAAGAACTGGGACATAGATATA;

ZD-672(K)-: TATATCTATGTCCCAGTTCTTATAACCATTTGGGTTAG;

ZD-672(R)+: CTAACCCAAATGGTTATCGCAACTGGGACATAGATA;

ZD-672(R)-: TATCTATGTCCCAGTTGCGATAACCATTTGGGTTAG.

### *In vitro* transcription and RNA transfection

All EV71 cDNA constructs were digested with *Hind*III to produce linear DNA template, purified by phenol/chloroform extraction, ethanol precipitation and then dissolved in RNase-free water. For *in vitro* RNA transcription reaction, 1 μg of linear DNA template was transcribed by MEGA script high yield transcription T7 kit in total volume of 20 μl. The *in vitro* transcripts were then purified by phenol/chloroform and dissolved in RNase-free water. 2 μg transcribed RNA was transfected into 2×10^5^ Vero cells using lipofectamine 2000. Clone-derived viruses were then passaged in RD cells to increase virus titers and stored at -80°C for subsequent experiments.

### Virus preparation and determination of virus titers

Virus supernatants were harvested when above 75% infected cells showed cytopathic effect. Cell debris was removed by centrifugation at 5,000g for 10 minutes, and then the supernatants were filtered through a 0.45 μm filter. The titer of virus stocks was determined using a TCID_50_ assay by the Reed and Muench method.

### Plaque assay

Virus plaque morphologies were determined by plaque assay as described previously [39]. Briefly, approximately 2×10^5^ Vero cells per well were seeded in a 6-well plate 24 hours in advance. A series of 1:10 dilutions were made by mixing 25μl of virus sample with 225 μl of DMEM medium. 200 μl of dilutions of viral supernatant were inoculated to individual well of 6-well plate. The plates were incubated at 37°C with 5% CO_2_ for 1 hour with being shaken every 15 minutes, and then the virus inoculum were replaced with 1 ml of DMEM medium containing 1% agarose. After 7 days of incubation at 37°C with 5% CO2, the agarose layer was covered with 1 ml of 1% agarose containing 0.01% neutral red to stain live cells at 37°C for4 hours in the absence of light. After staining, the number and the diameter of plaques were measured.

### Antibodies and Western blot assays

MAB979, a mouse monoclonal antibody that specifically recognizes EV71 VP0/VP2 capsid proteins (Millipore), MAB1255-M08, a mouse monoclonal antibody that specifically recognizes EV71 VP1 capsid protein (Abnova), and anti-β-actin antibody (Sigma-Aldrich) were used as primary antibodies. Infected cells or concentrated virus samples were lysed in loading buffer and resolved by electrophoresis in a 12% SDS-PAGE. Proteins were transferred onto a PVDF membrane, blocked with 5% skim milk, and probed with primary antibodies, including anti-EV71 VP0/VP2 antibody (diluted 1:1000), anti-EV71 VP1 antibody (diluted 1:1000), or anti-β-actin antibody (diluted 1:5000) at 4°C overnight. After incubation, the 1:5000 diluted HRP-conjugated secondary antibody was added, followed by incubation for 2 hours at room temperature. After four wash steps with PBS plus 0.1% Tween 20 (PBST), signals were detected using Western Lighting chemiluminescence reagent (Millipore) and the densitometry of specific blots was calculated using the ImageJ software.

### Neutralization blocking assay

Vero cells were seeded in 6-well plates 1 day prior to the assay. 2 μg *in vitro* transcribed viral genomic RNAs were transfected into cells using lipofectamine 2000. 2 hours post transfection, the antibody MAB1255-M08 (neutralization concentration of 1 μg/ml) was added into the medium, followed by 48 hours incubation.

### Cell-ELISA

The virus binding assay was performed using cell-ELISA. In brief, Vero cells were seeded in 96-well plates at a density of 10^4^ cells per well1 day prior to virus infection. 100 μl of virus dilutions were added to the cells. After 1 hour of incubation at 4°C for simultaneous adsorption, the unbound viruses were removed by three wash steps with PBS. The cells were then fixed in the wells with 4% paraformaldehyde for 20 minutes at room temperature, followed by blocking with 5% skim milk-PBS. The antibody solution (1:1000 EV71 VP1 monoclonal antibody, MAB1255-M08) prepared in PBS containing 5% skim milk was added into each test well and incubated for 1 hour at 37°C. After four times washing with PBST, HRP-conjugated anti-mouse IgG secondary antibody (1:5000 dilution) was added into each well and incubated for 40 minutes at room temperature. TMB peroxidase substrate was added and allowed to react for 30 minutes at room temperature. The reactions were stopped by adding 0.5M H_2_SO_4_. The absorbance was recorded at OD 450 nm using a microplate reader.

### Flow cytometry

The ability of EV71 binding to the cells was also determined by flow cytometric analysis. A total of 2×10^6^ suspended Vero cells in PBS were incubated with 300 μl virus supernatant for attachment at 4°C for 1 hour. After fixation by 1% paraformaldehyde, the cells were washed twice by PBS and re-suspended in 100 μl anti-EV71 VP1 antibody solution (1:500, Abnova). After incubation at 4°C for 1 hour, cells were washed four times by cold PBS containing 2% bovine serum, and then stained with FITC-conjugated anti-mouse IgG secondary antibody diluted at 1:100 and incubated on ice for 1 hour. After three washes with PBS, the stained cells were re-suspended in PBS, and the signals were detected using a Guava easyCyte flow cytometer and analyzed using the Guava ExpressPlus Assay.

### Quantification of virions by measuring viral genome RNA

For viral RNA quantification, 250 μl of the purified virus sample was used for RNA extraction by TRIzol LS (Invitrogen) according to the manufacturer’s instruction. cDNA was synthesized with reverse transcriptase of Moloney murine leukemia virus (Takara), and its level was quantified by real time PCR (Sso Fast Eva Green Supermix, Takara) using the primers EV71-RT-F (5’-gcagcccaaaagaacttcac-3’) and EV71-RT-R (5’-atttcagcagcttggagtgc-3’) that are located in the capsid protein VP1 gene. The copy number of the viral genome RNA was calculated by generating a standard curve using serial dilutions of plasmid containing DNA sequence for the VP1 gene.

### Genomes/capsid proteins ratio in EV71 virions

EV71 supernatants were purified by ultracentrifugation at 37,000 rpm for 2.5 hours at 4°C in a Beckman SW41Ti rotor through a 15% sucrose cushion. Protein concentrations of the samples were measured using the Bradford assay. Viral genomic RNA copies were determined by real time RT-PCR as described above. Viral genome copy numbers were expressed as copies/ml for each sample, and were then divided by the protein concentration (μg/ml), the final values represent the genome/capsid ratios.

### Quantification of negative strand genomic RNA

Vero cells were seeded in 6-well plates 1 day prior to the assay. The cells were inoculated with equal amount of HP strain or CCA strain, followed by incubation at 4°C for 1 hour. After synchronized viral adsorption, the cells were washed twice with PBS to remove the unbound virions. Then the cells were incubated at 37°C in the medium of DMEM. The virus infected cells were collected by Trizol reagent at indicated time points post infection. For detection of replication of negative strand genomic RNA, the total RNAs of the infected cell samples were extracted and subjected to RT-PCR quantification as described previously. In brief, cDNA was synthesized with reverse transcriptase of Moloney murine leukemia virus (Takara) using the primer 1F (5’-ttaaaacagcctgtgggttg-3’) specific to the negative strand genomic RNA. Then the cDNA of negative strand genomic RNA was quantified by real time PCR (SsoFast EvaGreen Supermix, Takara) using the primers EV71-RT-F (5’-gcagcccaaaagaacttcac-3’) and EV71-RT-R (5’-atttcagcagcttggagtgc-3’) that are located in the capsid protein VP1 gene. The gene of *GAPDH* was used as the internal control to normalize the total RNA of the sample. The negative strand RNA amount were expressed as fold increase relative to that of sample collected at 0.5 hour post infection.

### Internalization experiment

Vero cells were seeded in 6-well plates 1 day prior to the assay. The cells were inoculated with equal amount of HP strain or CCA strain, followed by incubation at 4°C for 1 hour. After synchronized viral adsorption, the cells were washed twice with PBS to remove the unbound virions. Virus internalization was initiated by incubation at 37°C for 30 minutes in the medium of DMEM containing 2% FBS. 100 μg/ml final concentration of subtilisin A was then added to the medium and incubated at 37°C for 15 minutes to remove the un-internalized virions. After digestion, PMSF was added to a final concentration of 5 mM to inactivate subtilisin A. The cells were lysed and subjected to western blotting analysis for detection of the VP1 protein.

### Sedimentation analysis

EV71 culture supernatant was harvested by three freezing-thawing cycles. The cell debris was removed by passing through a 0.45 μm filter, and then the supernatant was concentrated by centrifugation at 37,000 rpm for 2.5 hours at 4°C in a Beckman SW41 Ti rotor through a 15% sucrose cushion. The concentrated virions were further purified by a 15%-35% discontinuous sucrose gradient ultracentrifugation at 33,000 rpm for 70 minutes. After centrifugation, gradients were fractionated from the top in 0.5 ml aliquots. One half of each fraction was analyzed by RT-qPCR for quantification of viral genomic RNA and the rest was subjected to western blotting to detect viral proteins.

### Molecular dynamic (MD) simulations

VP1 protein configuration was extracted from the empty EV71 particle (PDB ID 3VBU). Residues 211–217 were missing in the PDB file. We added this loop before MD simulation. Four mutants (A107T, A107R, A107D and A107S) were generated using the Maestro software. A total of 10ns MD simulations were carried out for EV71-HP and mutants of VP1. MD simulations of EV71-HP and all these mutants of VP1 were performed using the Amber11 software package. The amber ff99SB force field was applied for the protein. TIP3P water molecules were utilized to solvate the complex, extending at least 10 Å from the protein. The counter ions (Cl-) were added for charge neutralization. To remove the bad contacts, the system was subjected to energy minimization. Firstly, the water molecules and ions were refined through 2,500 steps of steepest descent followed by 2,500 steps of conjugate gradient, keeping the protein fixed. Secondly, the whole system was relaxed by 10, 000 cycles of minimization procedure with 5,000 cycles of steepest descent and 5,000 cycles of conjugate gradient minimization. After that, the system was heated from 0 to 300 K by 500ps position restraint simulation. 2ns MD simulations without any restraints were sequentially performed to equilibrate the system. Finally, a length of 10 ns trajectory was computed at 300K under constant pressure. During the simulation, the cutoff distance for *van der* Waals interactions was set to 10.0 Å, along with a time step of 2.0 fs. Long-range electrostatic interactions are treated with the Particle Mesh Ewald (PME) method. The SHAKE algorithm was applied to constrain all bonds involving hydrogen atoms. All of the MD results were analyzed using the ptraj module of the Amber 11 software package.

### Silver-staining of proteins

The purified virus samples were lysed in loading buffer and resolved by electrophoresis in a 12% SDS-PAGE. After electrophoresis, the gel was fixed with 100 ml of the fixing solution (adding 50 ml of ethanol and 10 ml of acetic acid to 40 ml of ultrapure water) in a clean tray for 20 minutes or longer time. Then the gel was washed by 30% ethanol solution and ultrapure water in sequence. After that, the gel was stained with the ProteoSilver Plus Silver Stain Kit (Sigma) according to the manufacturer’s instructions.

### Statistical analysis

Unpaired two-tailed Student’s tests or one-way analysis of variance with the Tukey posttest were conducted to compare results obtained from the different experimental groups. *P* value of < 0.05 was considered statistically significant. In all figures, error bars indicate the standard deviations for 3 independent samples. Three asterisks indicate a *P* value lower than 0.01.

## Supporting information

S1 Fig CPE was monitored for EV71-CCA at 7 days post transfection. (TIF)

S2 Fig Quantification of viral genomic RNA and capsid protein of EV71-HP and EV71-CCA virions. (TIF)

S3 Fig Silver-staining patterns of the 160S particles of EV71. The protein bands of VP0, VP1, VP2 and VP3 were indicated respectively.
(TIF)
