# Supplementary figures and images for: A highly conserved amino acid in VP1 regulates maturation of enterovirus 71
Source: PLoS Pathog. 2017 Sep 22;13(9):e1006625. doi: 10.1371/journal.ppat.1006625 (PMC5634653; doi:10.1371/journal.ppat.1006625)

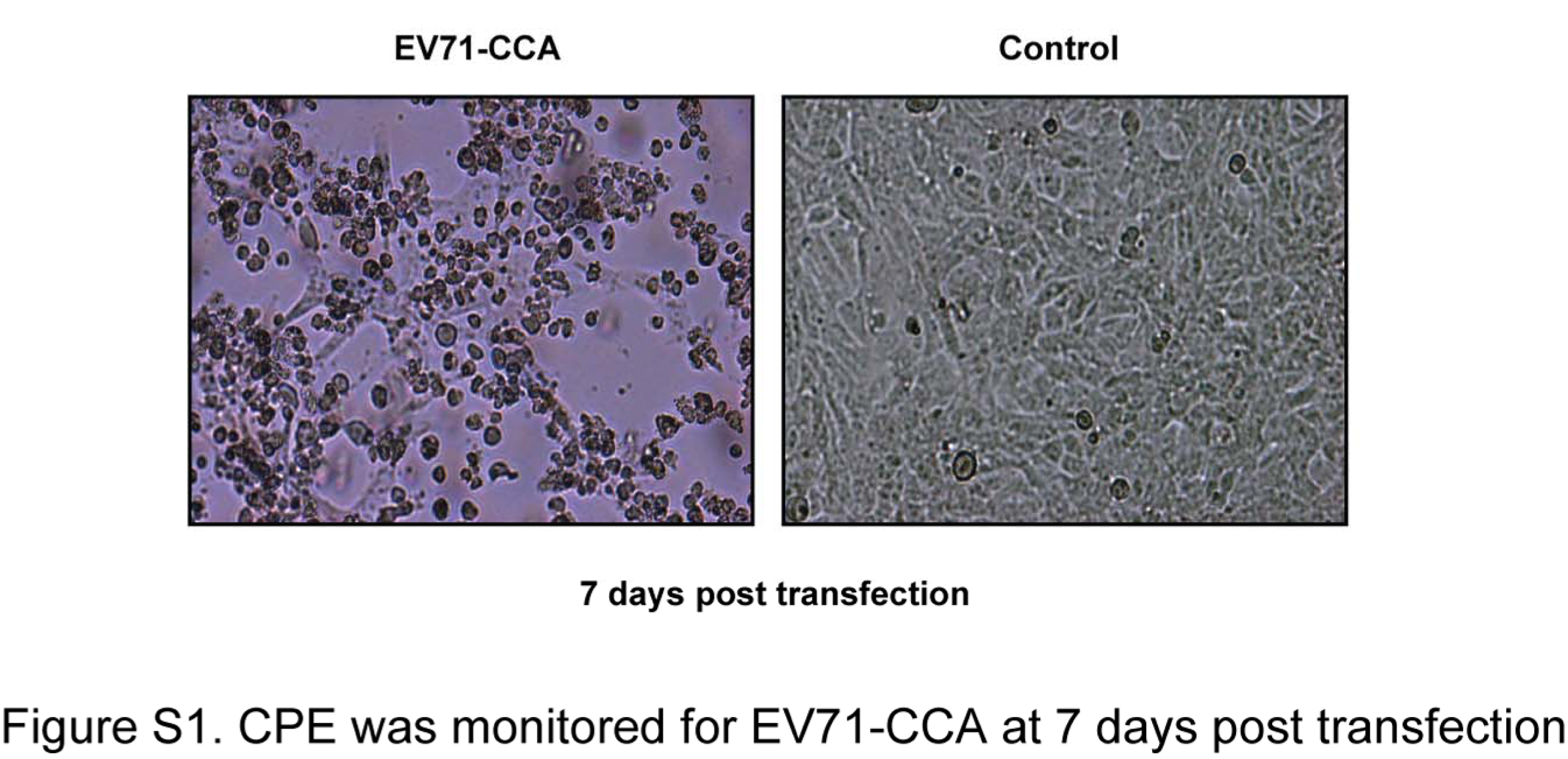

Supplement: S1 Fig — (TIF) [file ppat.1006625.s001.tif]

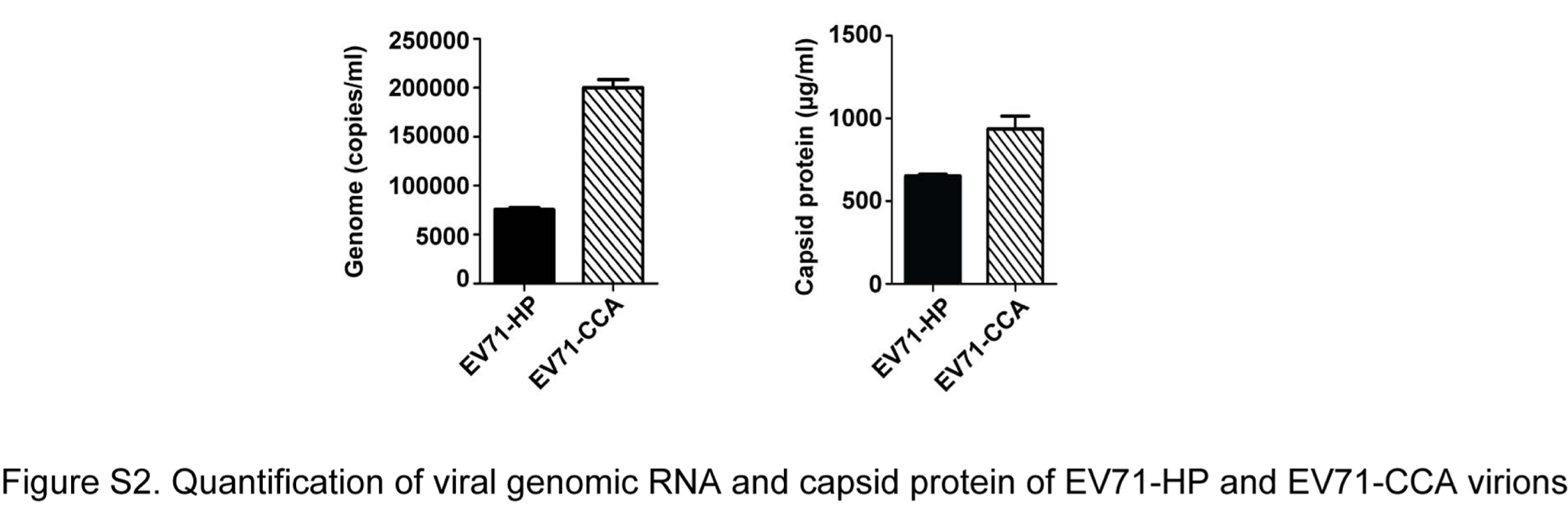

Supplement: S2 Fig — (TIF) [file ppat.1006625.s002.tif]

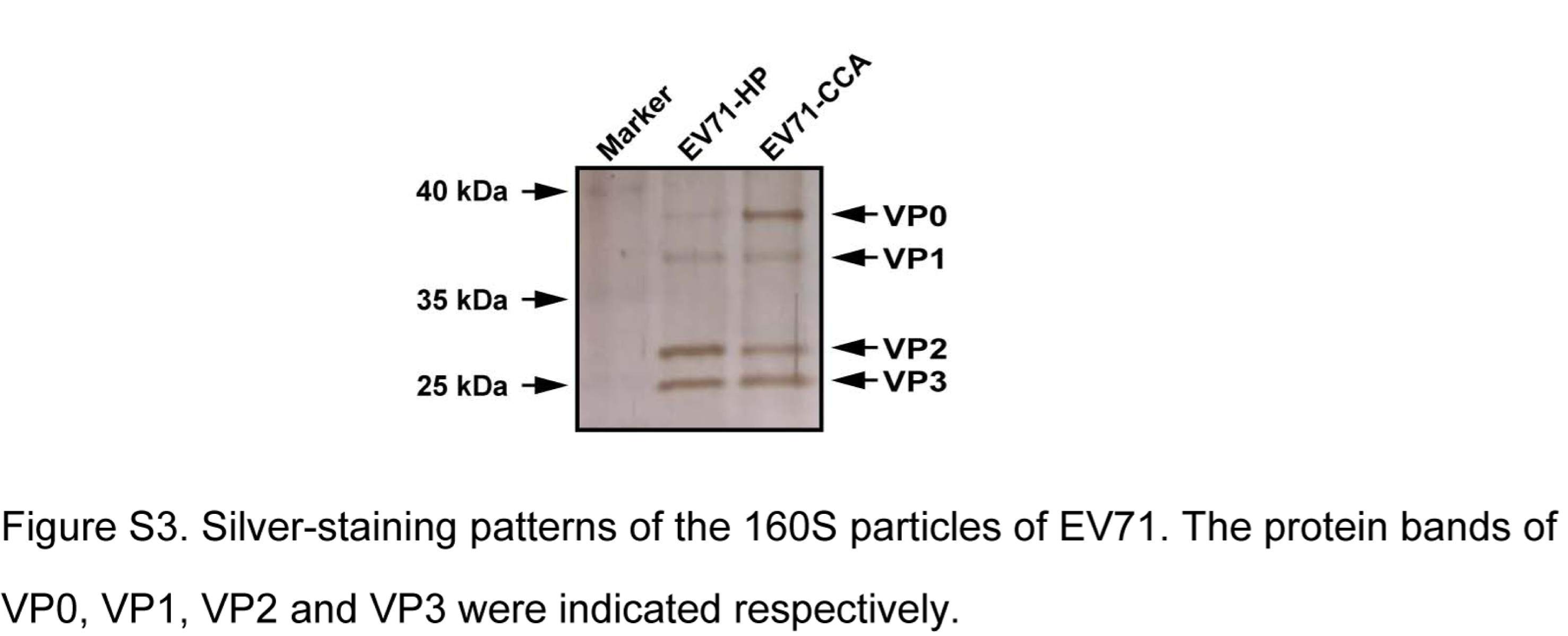

Supplement: S3 Fig — The protein bands of VP0, VP1, VP2 and VP3 were indicated respectively. (TIF) [file ppat.1006625.s003.tif]
